# The Challenge of Critical Feedback: A Simulation-based Advanced Residents as Teachers Module

**DOI:** 10.5070/M5.52392

**Published:** 2026-10-31

**Authors:** Haley Plattner, Elyse Fults, Thomas Alcorn, Sara M Hock

**Affiliations:** * Rush University Medical Center, Department of Emergency Medicine, Chicago, IL

## Abstract

**Audience:**

This is a simulation module intended to train residents at all levels in giving targeted, critical feedback to medical students on shift.

**Introduction:**

Residents as teachers (RAT) curricula are an important component of graduate medical education for residents who teach medical students.^1^ Developing residents’ skills in teaching, feedback, and supervision is therefore a critical component of training. Medical students receive a substantial portion of their clinical education from residents,^2^,^3^ and timely, specific feedback in the clinical setting improves learner performance and professional development.^4^ Despite the importance of these skills, residents have limited opportunities to practice delivering challenging feedback beyond introductory teaching experiences.^5^,^6^ This curriculum addresses that gap by providing simulation-based practice in identifying learner gaps and delivering targeted, critical feedback to medical students.

**Educational Objectives:**

The purpose of this module was to provide a safe learning environment for residents to practice giving critical feedback to medical students. By the end of the session, residents will be able to 1) identify gaps in student proficiency through analysis of a simulated student’s clinical performance as demonstrated by a simulated case presentation, 2) formulate specific feedback for student learners to target identified gaps, and 3) apply a newly learned strategy for providing feedback to a “student” during two simulation-based communication cases.

**Educational Methods:**

We developed two simulation communication cases wherein resident participants of all training years were placed in the roles of a simulated medical student and a simulated resident. Both roles are structured for resident participants only and the simulated student role was performed by another resident before the roles were then switched for the second case. This educational session was held in small groups during resident didactics with a facilitator present with each of the small group sessions. The first case involves a presentation of a patient with leg pain with an underlying diagnosis of necrotizing fasciitis, and the second involves a presentation of a patient with chest pain with an underlying diagnosis of pulmonary embolism. Face sheet information gives the simulated student and simulated resident unexpectedly different perspectives, leading the simulated student to omit can’t-miss diagnoses and requiring the resident to recognize an opportunity for feedback and practice delivering teaching points. The simulated student, played by another resident, is also invited to provide comments to their partner on the teaching strategies used during a facilitator-led debrief following the completion of each case

**Research Methods:**

Twenty-one participants completed pre-and post- surveys to gauge comfort with teaching and providing feedback to medical students on shift. Participants ranked their perceived importance of a “Residents as Teachers” curriculum in their medical education. We used a paired t-test to assess the change in rating before and after the cases. Statistical calculations were done using Excel. Participants provided qualitative data through open-ended feedback comments.

**Results:**

Resident confidence in teaching medical students on shift increased from 3.65 to 3.9 (*P*=0.01), and confidence in giving critical feedback increased from 3.2 to 3.95 (*P*<0.001). The perceived importance of a “Residents as Teachers” curriculum was overall unchanged from 4.4 to 4.5 (*P*>0.2). Review of feedback comments demonstrated an overall positive experience and several requests to receive further training on effective teaching. One resident playing the role of a “student” shared that they felt it was particularly helpful to play the role of the student to understand their perspective better, and several comments included a request for further opportunities for practice in the future. One resident suggested providing additional teaching on how to approach teaching students of different levels of training

**Discussion:**

The utilization of these modules was well-received by participants and proved to be an effective way to improve resident comfort in providing critical feedback and teaching medical students on shift. Providing the simulated student and simulated resident with divergent clinical perspectives allowed for a nuanced discussion regarding how the residents felt in their individual roles and their perceived efficacy of the feedback provided to the simulated student. Residents communicated that the cases were reflective of experiences they have had on shift and appreciated the ability to practice their skills in a safe environment. They reported overall satisfaction with the cases and a desire to use similar cases in the future.

**Topics:**

Simulation, medical education, residents as teachers curriculum, feedback.

## USER GUIDE

**Table:** 

List of Resources:	
Abstract	153
User Guide	155
Small Groups Learning Materials	159
Appendix A: Facilitator Guide to Cases	161
Appendix B: Simulated Resident Cases	164
Appendix C: Simulated Student Cases	166

**Learner Audience:**

This simulation module is designed for medical students, interns, and junior and senior residents.

**Time Required for Implementation:**

This session can be implemented with approximately 45 min of in-person simulation time per learner group.

**Recommended Number of Learners Per Instructor:**

We recommend groups of 2–4 learners depending on resource availability and time constraints.

**Topics:**

Simulated participant, residents as teachers, RAT curriculum, feedback.

**Objectives:**

By the end of this session, learners will be able to:

1. Identify gaps in student proficiency through analysis of a simulated student’s clinical performance as demonstrated by a simulated case presentation.
2. Formulate specific feedback for student learners to target identified gaps.
3. Apply a newly learned strategy for providing feedback to a “student” during two simulation-based communication cases

### Linked objectives and methods

Each residency program is required to have incorporated a residents as teachers curriculum in place,^1^ and this educational session is designed to follow an introductory residents as teachers curriculum as an opportunity for spaced repetition. This curricular component is intended to be a simulation-based option for residency programs seeking further practice in an applied setting. A systematic review by Hill et al found that roughly half of studied institutions used one-off resident-as-teacher interventions, and roughly a third used seminar- or lecture-style sessions; limited opportunities for feedback practice were also identified.^5^ Within emergency medicine, more focused curricula may incorporate role play and simulation to improve residents’ teaching skills.^10^ Published guidance for resident-as-teacher curricula recommends incorporating adult learning theory, fostering a positive learning environment, establishing clear learning objectives, and teaching bedside instruction and feedback techniques.^11^,^14^ A large proportion of studies identified in the literature review utilized the One-Minute Preceptor model^14^ as a common useful method for providing on-shift teaching skills; this model was incorporated into the present curriculum as a structured framework for bedside teaching. Initially proposed for use by educators in a busy clinical environment, the model and focuses on 5 microskills: “(1) get a commitment, (2) probe for supporting evidence, (3) teach one general rule, (4) reinforce what was done right, and (5) correct mistakes”.^6^,^7^ Additionally, Pendleton’s method of feedback, a structured method for learners to self-assess their performance before receiving additional feedback provided by the educator, was included because of its versatility across both classroom and clinical settings, its ability to encourage buy-in from learners, as well as its ability to promote constructive feedback while reinforcing positive learner behaviors.^8^

Repeated practice of delivering challenging feedback leads to improved skill, and limited further case formats for practice exist. But a gap was identified in practice opportunities at an advanced level for delivering critical feedback to medical students. As recognized by Funey et al^6^ and Hill et al,^5^ limited practice opportunities exist for feedback experiences. This curriculum addresses that gap and provides a curricular element that can address the deficiency.

### Objective 1: Identify gaps in student proficiency through analysis of a simulated student’s clinical performance as demonstrated by a simulated case presentation

During this learning module, residents participate as a simulated student or a simulated resident and are given a face sheet containing information about the example case. In designing these cases, the authors identified several realistic differences in perspective that may occur in a medical student-resident interaction. In case 1, the simulated student is provided a face sheet that provides the information needed to present a case about a patient with right leg pain to the simulated resident. However, the “student” face sheet omits vital signs from the presentation and induces the simulated student to provide a differential and plan focused on cellulitis. The simulated resident face sheet provides the patient’s abnormal vital signs and risk for necrotizing fasciitis. The differing perspectives prompt the resident to ask further questions of the simulated student to determine the gaps in the “student’s” presentation and differential. In case 2, the participants switch roles. The simulated student reads aloud a presentation on a patient with chest pain that contains too much information and dictates a pause after the history and physical. This requires the simulated resident to ask further questions and prompts the “student” to provide an assessment and plan which provides a differential for chest pain that does not include pulmonary embolism, simulating a gap in the “student’s” recognition of a common, life-threatening differential in a high-risk patient. In both cases, the content creates simulated gaps for the resident to identify.

### Objective 2: Formulate specific feedback for student learners to target identified gaps

Simulated residents provide feedback to the simulated student in the first scenario using the residents’ pre-existing skills in the simulated environment. This allows the residents to identify opportunities for improvement in teaching skills and provides an observable baseline in teaching skills to help the facilitator best target their debrief to the specific learners. Following the initial simulated teaching session, the simulated resident and simulated student are invited to discuss their experiences with the facilitator in a debriefing session. Facilitators were expected to observe the residents during the cases and provide feedback on the simulated resident’s recognition of the simulated student’s gaps and their approach to teaching and feedback that was provided in response to those gaps. Discussion also included communication style and feedback frameworks. The authors also found it particularly valuable for the simulated student to experience a realistic but unexpected difference in perspective during the case to allow them to provide feedback to the simulated resident on the way in which they helped the “student” understand their error.

### Objective 3: Apply a newly learned strategy for providing feedback to a “student” during two simulation-based communication cases

Subsequent to initial feedback after the first case is completed, the facilitator offers a review of the one-minute-preceptor model and Pendleton’s method for feedback. The participants then switch roles, and the simulated resident is coached to use either of these models during the second case. Following the conclusion of the simulated resident feedback case, faculty facilitators then lead a debriefing conversation which includes feedback on the implementation of the new method for the simulated resident.

### Recommended pre-reading for facilitator

Facilitators should be familiar with general instructional methods to include the one-minute preceptor model,^6^,^7^ Pendleton’s method for feedback^8^,^9^ and other RAT curricular elements.^1^,^10^,^11^,^12^,^13^ Descriptions of the one-minute preceptor model and Pendleton’s method for feedback have been included in the facilitator guide, and facilitators are expected to provide a brief description of both models during the debrief portion following the cases.

### Learner responsible content (LRC)

Learners should be prepared through an introductory residents-as-teachers curriculum that takes place at each institution that has medical students. Junior learners who have not yet encountered the initial curriculum as part of their didactic experiences could be offered a student perspective on the importance of resident teachers or the associated commentary blog and video.^2^,^16^

### Small group application exercise (sGAE)

See the following attached materials for this small group exercise

- Appendix A: Facilitator Guide to Cases
- Appendix B: Simulated Resident Cases
- Appendix C: Simulated Student Cases

### Results and Tips for Successful Implementation

Twenty-one residents participated in the communication cases in small group sessions no bigger than 4–6 in size. For the first case, 2 residents were assigned roles of the simulated student and simulated resident, and for the second case, the roles were assigned to 2 different residents. The cases are written to be appropriate for both senior and junior residents for both roles, provided they have had at least an introductory Residents as Teachers session beforehand. Junior residents who have not yet had an introductory training could benefit from taking the role of the simulated student to learn from the perspective of a medical student receiving teaching and feedback on shift. The participants were given a 4-question pre-survey and a 5-question post-survey with the ability to provide general feedback in the post-survey. One hundred percent of the participants filled out the pre- and post-surveys. Resident confidence in teaching medical students on shift increased from an average of 3.65 to 3.90 (*P*=0.01), and confidence in giving critical feedback increased from an average of 3.20 to 3.95 (*P*<0.001). The perceived importance of a “Residents as Teachers” curriculum was overall unchanged from 4.40 to 4.50 (*P*>0.2), indicating a strong pre-existing perceived importance of this curriculum to residents. The written comments provided in the post-survey were overwhelmingly positive regarding the communication cases and consistently communicated a desire to receive even further training on how to be effective teachers. Representative comments include, “I thought the discrete learning points discussed after were helpful and something I can take to my next shift.,” “Great way to prepare in a non-intimidating way, made better by having real cases,” and “Always good to have a refresher. Makes it easier to actually enact on-shift.”

Several comments provided feedback that they wish their didactic curriculum included sessions like this more often to provide repeated practice and opportunities to practice with more variety of case presentations and teaching methods.

Facilitators should also be prepared to observe behaviors of the simulated resident, including their ability to recognize gaps in the simulated student’s knowledge and understanding, and their communication skills with teaching and providing feedback, both during the simulation cases and during the debrief. They are recommended to have reviewed both the One-Minute Precepter model and Pendleton’s method for feedback prior to facilitating the small groups. In this study, the facilitators were provided a handout on both methods to then teach to the participants during the debrief. In addition, the facilitators were provided a debriefing guide that included suggestions of questions to ask following the PEARLS debriefing framework.^15^

It was observed by the facilitators that larger groups were more difficult because there were only 4 roles available for participation per group between the two cases.

We suggest the following strategies for successful implementation:

- These sessions are best done with no more than 4 participants per group and 1–2 faculty facilitators to allow all to participate in some way, either as a recipient of feedback, providing feedback or both.
- The sessions can be done anywhere, but we suggest having them in a quiet room with only a small group in order to provide a safe environment for participants to try new methods of feedback and discuss how the cases made them feel.
- Prepare the participants ahead of time to follow the cases as presented on their face sheets because sometimes the simulated student would naturally try to alter the provided information to provide a more polished presentation. We found they were more likely to provide case presentations as prompted by the face sheet when they were able to understand their role better and were prepared ahead of time to have some missed information in their cases.

### Pearls

By the end of the session, learners should know how to use the one-minute preceptor model and Pendleton’s method in providing teaching/feedback to medical students on shift. They should be able to identify learning gaps in these medical students and be able to provide targeted feedback to the specific gap in the student’s medical knowledge. One-Minute Preceptor Model:^6^,^7^

The 5 Microskills

1. Get a Commitment. Ask the learner for their own assessment and plan before offering yours. This pushes them to reason independently rather than passively receiving answers. Use “what” questions: “What do you think is going on?”
2. Probe for Supporting Evidence. Ask “why” questions to uncover the learner’s clinical reasoning: “Why would you order a complete metabolic panel on this patient?” This step ensures you understand their thought process and can identify knowledge gaps.
3. Teach One General Rule. Provide a generalizable pearl or principle the learner can apply to future encounters. The focus is on a single, transferable teaching point rather than an exhaustive lecture.
4. Reinforce What Was Done Well. Provide specific positive feedback about what the learner did correctly. Being specific is key -- “You did a great job talking with that patient’s family” rather than a vague “good job.”
5. Provide Coaching: Correct any mistakes. Point out errors or areas for improvement using specific, actionable language. Correcting mistakes away from the patient and public view is recommended. Discuss strategies for improvement.

Pendleton’s Method for Feedback:^8^,^9^

The Five Rules

1. The feedback provider (FBP) asks the feedback recipient (FBR) to mention 2–3 points that went well.
2. The FBP shares 2–3 points that went well.
3. The FBP asks the FBR to mention 2–3 points that can be improved.
4. The FBP shares 2–3 points for improvement and discusses strategies to close the gap.
5. Either the FBP or FBR summarizes the most important points from the conversation.

## SMALL GROUPS LEARNING MATERIALS

### Small Group Application Exercise (sGAE)

This exercise is designed as an approximately 40-minute, in-person small-group session. Divide learners into groups of 3–4 residents, with no more than 4 participants per group when possible and assign 1 faculty facilitator to each group; a second facilitator may assist if resources permit. Conduct the session in a quiet room or other separate space that allows participants to practice teaching and feedback and discuss their reactions in a psychologically safe setting.

Before the session, facilitators should review the One-Minute Preceptor model, Pendleton’s method for feedback, and the PEARLS healthcare debriefing framework. Prepare the simulated student and simulated resident instruction sheets for both cases, the facilitator guides, and the teaching handouts for the One-Minute Preceptor model and Pendleton’s method. At the beginning of the small group, explain that the activity consists of two 10-minute communication cases followed by 10 minutes of facilitated debriefing. Also explain during the pre-brief that the simulated student and simulated resident will receive intentionally different information, since the student materials contain purposeful omissions or imperfect presentations. Participants should follow the information on their assigned face sheets as written rather than adding, correcting, or polishing information.

For Case 1, assign one resident to the simulated student role and one resident to the simulated resident role and any remaining learners observe. The simulated student presents the case from the provided sheet, and the simulated resident responds with teaching and feedback using their pre-existing approach. After the case, conduct a PEARLS-guided debrief and review the One-Minute Preceptor model.

For Case 2, rotate roles so that different residents serve as the simulated student and simulated resident when group size permits. The simulated resident should apply a feedback strategy introduced during the first case debrief. After the second case, repeat the PEARLS-guided debrief and review Pendleton’s method for feedback. In a group of three, one learner may need to participate in an active role in both cases so that both cases can be completed.

**Debrief Method [using the PEARS healthcare debriefing tool]** 15

Reactions phase:

- “How did you feel about the case?”
- “What went well, and what did not go well?”
- Invite the simulated student to provide additional feedback on how the interaction felt.

Description phase

- “What were the knowledge/understanding gaps that the ‘student’ had in this case?”

Analysis Phase

- “This ‘student’ had several possible teaching points; how do you prioritize what you will teach about on shift?”
- “How have you dealt with similar scenarios with on-shift teaching in the past?”

Application Phase

- “ What is one thing you learned today that you will apply the next time you are teaching a medical student on-shift?”

## Appendix A Facilitators Guide to Cases

**Case 1 Facilitator Instructions:**

Use the Simulated Student Case 1 and Simulated Resident Case 1 instruction sheets. The simulated student presents a patient with right leg pain, while the simulated resident has access to abnormal vital signs and additional information that increases concern for necrotizing fasciitis. The resident should identify the important omissions or discrepancies and provide targeted teaching and feedback.

Objectives for the simulated resident role:

- Identify gaps in student proficiency through analysis of a student’s clinical performance as demonstrated by a simulated case presentation.
- Formulate specific feedback for “student” learners to target identified gaps.

**Case 1 specific facilitator focus:**

- The student presentation intentionally omits the vital signs and emphasizes cellulitis; observe whether the resident recognizes the discrepancy and probes for additional information.
- Observe how the resident prioritizes the student’s knowledge gaps and whether the feedback is specific and actionable.
- After the case-specific discussion, use the session-wide debrief structure above and present the One-Minute Preceptor Model.

One-Minute Preceptor Model:^6^,^7^

The 5 Microskills

1. Get a Commitment. Ask the learner for their own assessment and plan before offering yours. This pushes them to reason independently rather than passively receiving answers. Use “what” questions: “What do you think is going on?”
2. Probe for Supporting Evidence. Ask “why” questions to uncover the learner’s clinical reasoning: “Why would you order a complete metabolic panel on this patient?” This step ensures you understand their thought process and can identify knowledge gaps.
3. Teach One General Rule. Provide a generalizable pearl or principle the learner can apply to future encounters. The focus is on a single, transferable teaching point rather than an exhaustive lecture.
4. Reinforce What Was Done Well. Provide specific positive feedback about what the learner did correctly. Being specific is key -- “You did a great job talking with that patient’s family” rather than a vague “good job.”
5. Provide Coaching: Correct any mistakes. Point out errors or areas for improvement using specific, actionable language. Correcting mistakes away from the patient and public view is recommended. Discuss strategies for improvement.

**Case 2 Facilitator Instructions:**

Use the Simulated Student Case 2 and Simulated Resident Case 2 instruction sheets. The simulated student presents a patient with chest pain and prior deep vein thrombosis, gives an overly detailed presentation, pauses before the assessment and plan, and omits pulmonary embolism from the differential. The resident should identify these gaps and apply a feedback strategy introduced during the first case debrief.

Objectives for the simulated resident role:

- Identify gaps in student proficiency through analysis of a simulated student’s clinical performance as demonstrated by a simulated case presentation.
- Formulate specific feedback for student learners to target identified gaps.
- Apply a newly learned strategy for providing feedback to a “student” during two simulated-based communication cases.

**Case 2 specific facilitator focus:**

- Observe whether the resident recognizes the missing high-risk diagnosis, redirects the presentation appropriately, and elicits an assessment and plan when the student pauses.
- Observe how the resident applies the newly learned feedback strategy and whether the feedback is specific and actionable.
- After the case-specific discussion, use the session-wide debrief structure above and present Pendleton’s Method for Feedback.

Pendleton’s Method for Feedback:^8^,^9^

The Five Rules

1. The feedback provider (FBP) asks the feedback recipient (FBR) to mention 2–3 points that went well.
2. The FBP shares 2–3 points that went well.
3. The FBP asks the FBR to mention 2–3 points that can be improved.
4. The FBP shares 2–3 points for improvement and discusses strategies to close the gap.
5. Either the FBP or FBR summarizes the most important points from the conversation.

## Appendix B Simulated Resident Cases

**Simulated Resident Case 1:**

You are the resident on a case supervising a medical student during a busy evening shift. You have sent the student to evaluate a patient with a chief complaint of right leg pain. In the meantime, you have had the chance to review the vitals, triage note and chart review. Your patient is a 57-year-old male with a past medical history of diabetes mellitus and hypertension who presents with right leg pain for 3 days and fever for 1 day.

**Table:**

Vitals:	Blood pressure: 94/56	Heart rate: 128	Respiratory rate: 28
	Temperature: 38.3 °C	Pulse oximetry: 93%	

While the student was evaluating the patient, the nurse came up to you and noted that he has a rash that has been tracking up his leg and is worse than even when he was first triaged 2 hours ago.

You are tasked with listening to patient presentations from the student and providing teaching and feedback as necessary.

**Table:**

Laboratory Risk Indicator for Necrotizing Fasciitis (LRINEC)^17^ Composite score: Low risk: <6; Intermediate: 6–7; High risk: ≥8		
CRP (mg/dL)	<15	0
	≥15	4
WBC (×1,000/μL)	<15	0
	15–25	1
	>25	2
Hemoglobin (g/dL)	>13.5	0
	11–13.5	1
	<11	2
Sodium (mEq/L)	≥135	0
	<135	2
Creatine	≤1.6 mg/dL or 141 μmol/L	0
	>1.6 mg/dL or 141 μmol/L	2
Glucose	≤180 mg/dL or 10 mmol/L	0
	>180 mg/dL or 10 mmol/L	1

**Simulated Resident Case 2:**

You are the resident on a case supervising a medical student during a busy evening shift. You have sent the student to evaluate a patient with a chief complaint of chest pain. In the meantime, you have had the chance to review the vitals, triage note and chart review.

Your patient is a 57-year-old female with a past medical history of diabetes mellitus, hypertension, and prior deep vein thrombosis who presents with chest pain and shortness of breath.

On chart review, you notice that she has had a history of prior deep vein thrombosis and is not currently on anticoagulation.

**Table:**

Vitals:	Blood pressure: 136/86	Heart rate: 115	Respiratory rate: 28
	Temperature: 37 °C	Pulse oximetry: 93%	

You are tasked with listening to the patient presentation from the student and providing teaching and feedback as necessary.

## Appendix C Simulated Student Cases

**Simulated Student Case 1:**

Your role is the medical student during a busy shift and you’ve been sent by the resident to evaluate a patient with chief complaint of right leg pain.

You gather the following information and formulate a plan as below. After presenting by reading the below paragraphs, respond as you believe a student would to the resident’s teaching points. After the scenario, you will be asked to **give feedback** about the teaching to your peer.

**Presentation**:

The patient is a 57-year-old male with a past medical history of diabetes mellitus and hypertension presenting with right leg pain. The pain started approximately 3 days ago and has been progressively worsening. He notes swelling and a red rash on the leg that started around the same time but got much worse today. He thinks he may have bumped his leg while working in his garden recently but reports no overt trauma to the area. He also has been feeling feverish today and mildly short of breath. No other associated symptoms, no chest pain, abdominal pain, nausea or vomiting. Physical Exam:
*[omit vitals: student forgot to write them down]*
Cardiopulmonary: no abnormalities Abdominal: no abnormalities Extremities: Right leg erythema and swelling. Tender to palpation. Good distal pulses and full range of motion Differential: Cellulitis, deep vein thrombosis, abscess, fracture, or dislocation Plan: Right lower extremity duplex ultrasound; however, most likely differential is cellulitis, so anticipate disposition to discharge with prescription for topical antibiotic: bacitracin.

**Simulated Student Case 2:**

Your role is the medical student during a busy shift and you’ve been sent by the resident to evaluate a patient with a chief complaint of chest pain.

You gather the following information and formulate a plan as below. After presenting by reading the below paragraphs, respond as you believe a student would to the resident’s teaching points. After the scenario, you will be asked to **give feedback** about the teaching to your peer.

**Presentation**:

The patient is a 57-year-old female with a past medical history of diabetes mellitus, hypertension, and prior deep vein thrombosis presenting with chest pain. The pain started approximately 6 hours ago and has been progressively worsening. She notes the pain is located over the left side of her chest, non-radiating, and associated with shortness of breath which is worse with exertion. The pain is described as a sharp pain and worse with coughing or taking a deep breath. No other associated symptoms, no leg pain or swelling, abdominal pain, nausea or vomiting. Medication history: Lisinopril 20 mg once daily Metformin 1000 mg twice daily Glipizide 10 mg daily Used to be on Eliquis, but not taking anymore Physical Exam: GENERAL APPEARANCE: Activity normal for age, well developed/well nourished, no cyanosis, pallor, or diaphoresis. EYES: lids/conjunctiva normal. EARS/NOSE/THROAT: Mucous membranes moist, nares normal, lips/teeth normal, uvula midline without oral pharyngeal erythema, exudate or swelling, tympanic membranes are normal bilaterally. No lymphangitis/lymphedema. HEAD/NECK: Normocephalic, atraumatic, no facial trauma, neck is supple. RESPIRATORY: Respiratory effort normal, speaks in full sentences, no tripod position, no accessory muscle use. Lungs clear to auscultation without rhonchi, wheezes, rales. CARDIAC: Tachycardia noted, no edema. ABDOMINAL: Soft, Non-distended, non-tender. No evidence of fluid wave. No pulsatile masses on exam, rebound tenderness, Murphy sign or pain over Mcburney’s point. MUSCLES/EXTREMITIES: No abnormal range of motion, no swelling. SKIN: Warm, pink and dry. No rashes, dermatoses, petechiae or lesions.
*Long pause here until prompted to move on to assessment and plan.*
Differential: Well, I’m not really sure other than acute coronary syndrome and acid reflux, maybe pneumonia? So for my plan, I would think: a complete blood count (CBC), complete metabolic panel (CMP), electrocardiogram, troponin, chest x-ray, and aspirin.
